# Supplementary material for: Neutrophils homing into the retina trigger pathology in early age-related macular degeneration
Source: Commun Biol. 2019 Sep 20;2:348. doi: 10.1038/s42003-019-0588-y (PMC6754381; doi:10.1038/s42003-019-0588-y)
Supplement: Supplementary file 1 — Supplementary Information [file 42003_2019_588_MOESM1_ESM.pdf]

Supplementary Figures

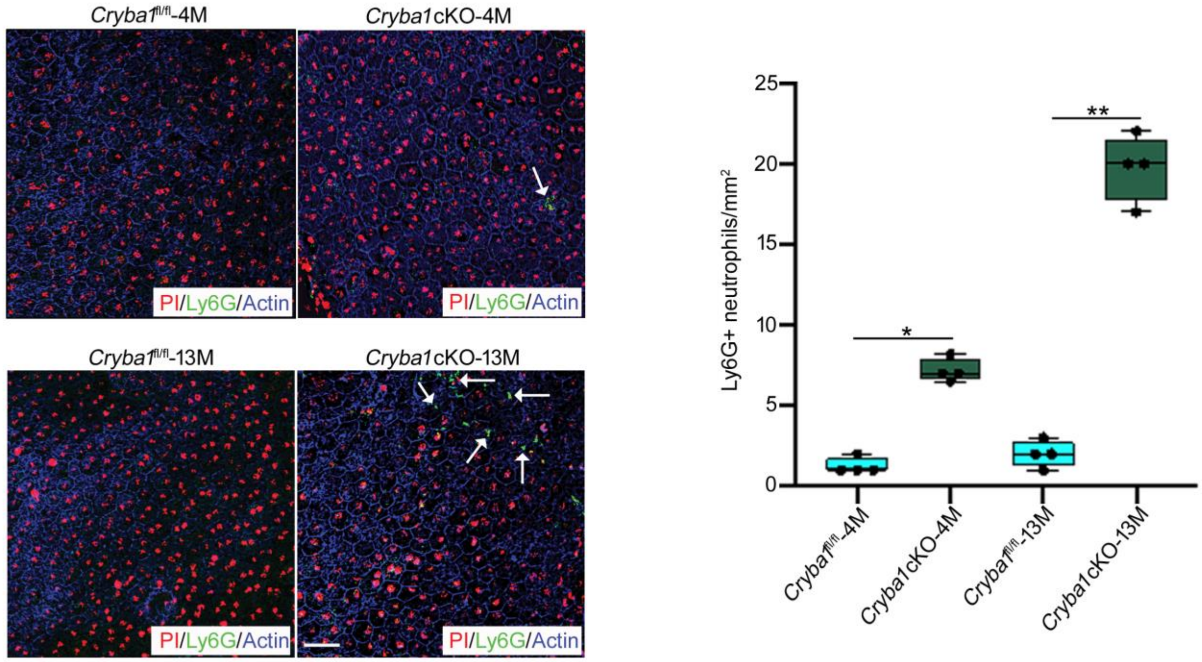

Supplementary Figure 1. Sub-retinal accumulation of neutrophils in *Cryba1* cKO mice. Immunofluorescence studies followed by quantification of Ly6G$^+$ cells (Green, Neutrophil marker) on RPE flatmounts, counterstained with propidium iodide (PI, Red, which stains nuclei) and actin (Blue) showed significant increase in Ly6G$^+$ neutrophils in *Cryba1* cKO mice as a function of age, relative to floxed controls (*Cryba1*$^{fl/fl}$). n=4. *P< 0.05, **P< 0.01 (One-way ANOVA and Tukey's post-hoc test). Scale bar, 50 μm.

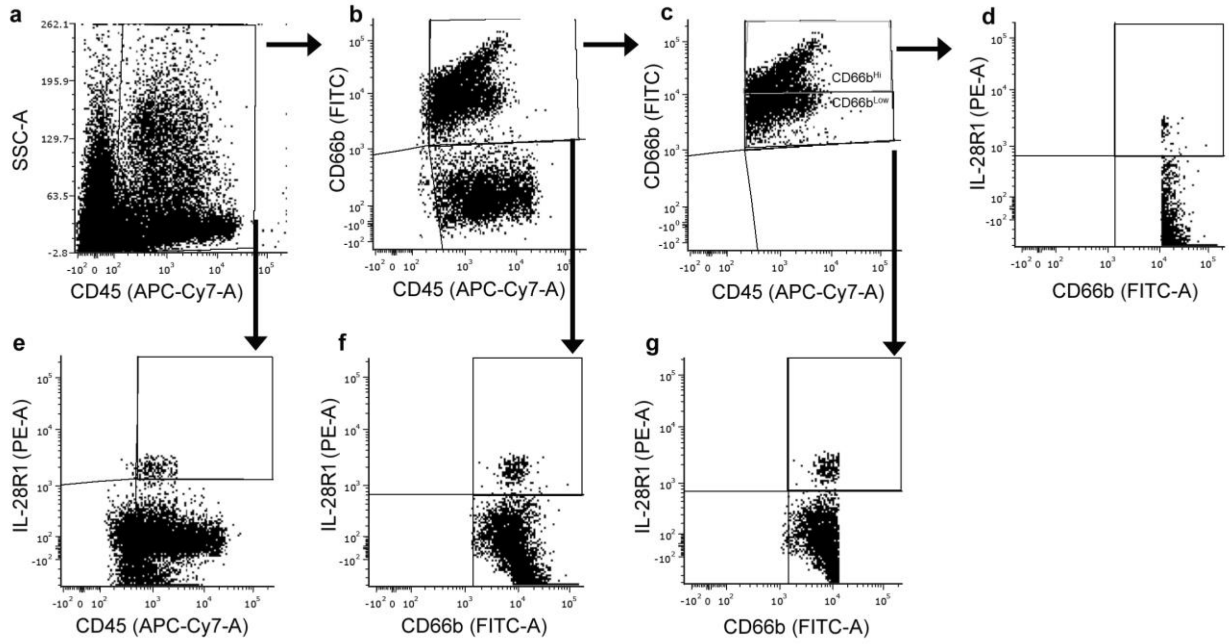

Supplementary Figure 2. Gating strategies for immune cell populations from human samples. Representative images showing gating strategies to evaluate the immune cell populations in the peripheral blood samples from control and AMD subjects. The cells from respective samples were stained for different cell surface markers and serial gating strategies (marked with black arrows) were performed among leukocytes ($CD45^+$ cells). (**a**) CD45 (APC-Cy7-A, leukocytes) vs Side Scatter (SSC-A), representing the leukocytes population in the different samples. The $CD45^+$ cells were further used to evaluate other cell populations in the samples. (**b**) $CD45^+CD66b^+$ (neutrophils) cells were gated from the total $CD45^+$ leukocytes from 'a'. (**c**) Representative gating denoting $CD45^+CD66b^{high}$ (activated neutrophils) and $CD45^+CD66b^{low}$ (naive neutrophils) population of cells among the $CD66b^+CD45^+$ neutrophils from 'b'. (**d**) $CD66b^{high}IL28R1^+$ (activated neutrophils expressing IFNλ receptor) cells were gated from the $CD66b^{high}$ population from 'c'. (**e**) Leukocytes expressing IFNλ receptor ($CD45^+IL28R1^+$) were gated from the total $CD45^+$ cells (in 'a') from each sample. (**f**) Neutrophils ($CD66b^+$ cells) expressing IFNλ receptor ($CD66b^+IL28R1^+$) were gated from the

total $CD45^+CD66b^+$ cells (in ‘b’) from each sample. (**g**) Naive neutrophils expressing IFNλ receptor ($CD66b^{low}IL28R1^+$) were gated from the $CD45^+CD66b^{low}$ cell population from ‘c’.

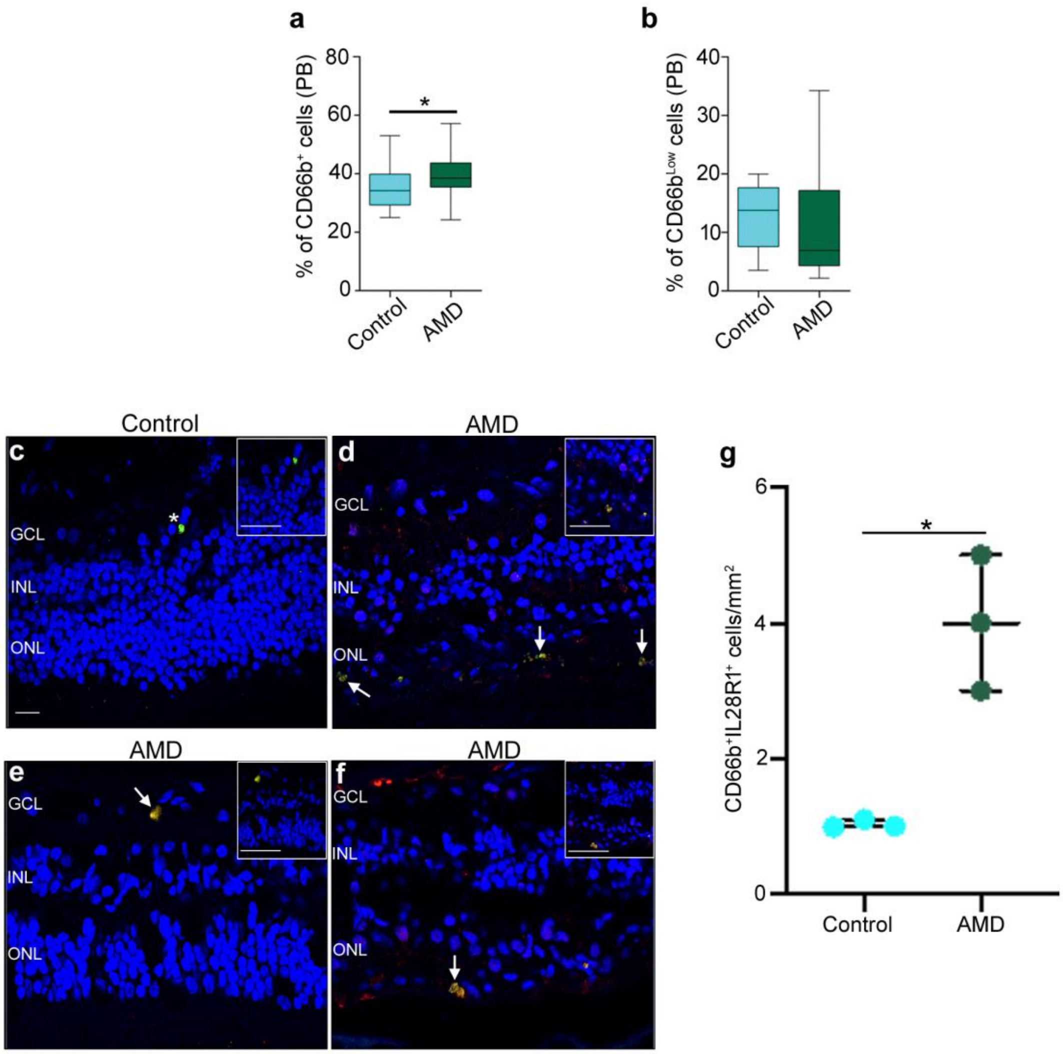

Supplementary Figure 3. Neutrophil status in peripheral blood and retina of early AMD human subjects. Flow cytometry analysis (gated as described in Supplementary Figure 2) showing significant change in (**a**) total CD66b^{+} cells (neutrophils) in peripheral blood (PB), of AMD patients compared to controls, with no significant change in the levels of CD66b^{low} cells (naïve neutrophils) (**b**). Peripheral blood (AMD; n=43 and Controls; n=18). **P*<0.05. Note: *P*-value for B: 0.06 (Mann-Whitney test). Immunofluorescence study followed by quantification for CD66b^{+} (neutrophils) IL28R1^{+} (IFNλ receptor) double positive cells, showed increased prevalence of CD66b^{+}IL28R1^{+} cells (arrows, inset showing zoomed image of the region of

interest) in retinal sections from human AMD patients (**d-g**), compared to control subjects (**c and g**) which showed a lower number of CD66b+ neutrophils with no expression of IL28R1 in these cells (asterisk). n=3. *P< 0.05 (One-way ANOVA and Tukey’s post-hoc test). Scale bar, 50 μm (Inset: 20 μm).

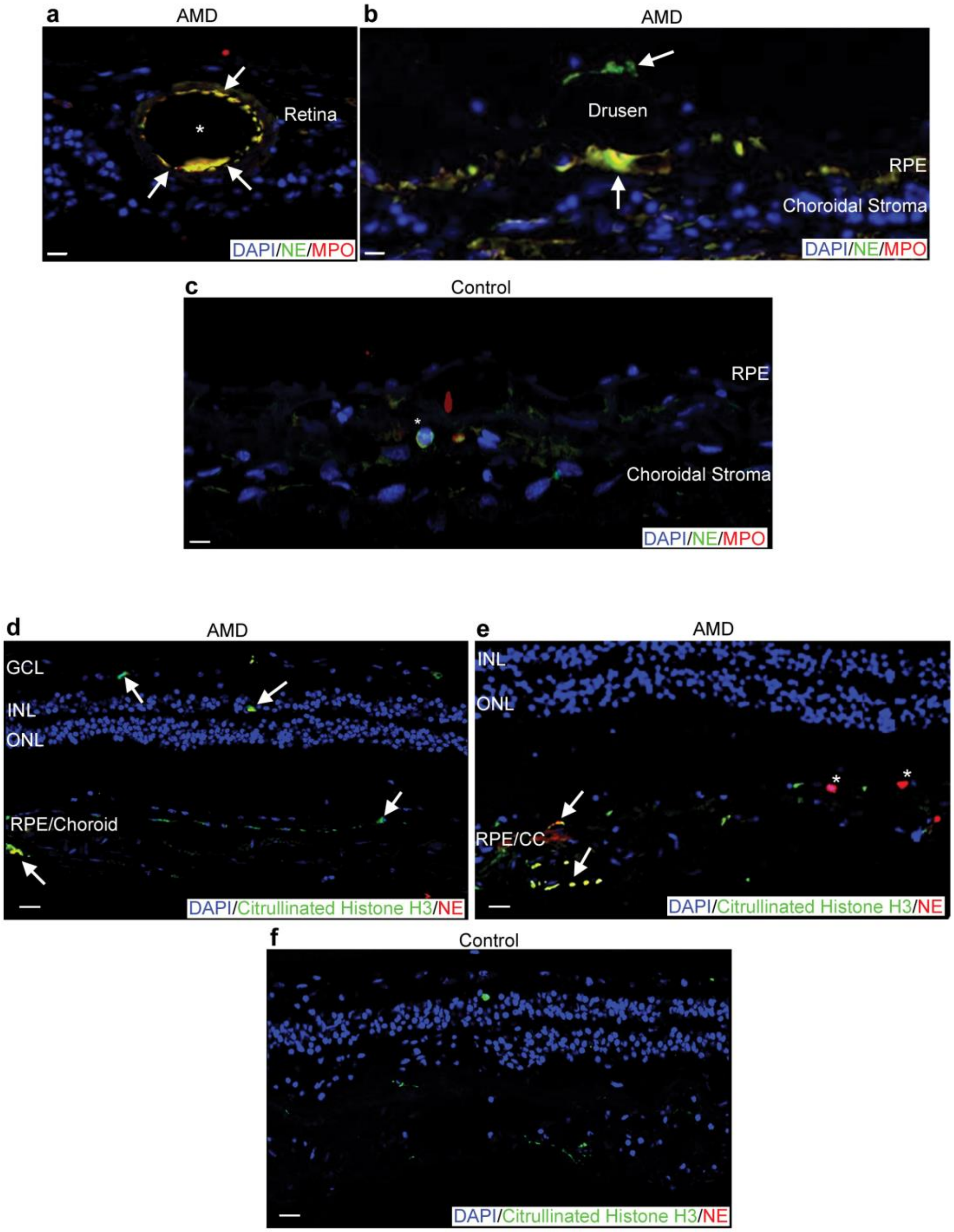

Supplementary Figure 4. Increased expression of neutrophil extracellular traps in infiltrating neutrophils in human AMD retina. NE (Neutrophil elastase, Green) and MPO (Myeloperoxidase, Red) immunostaining of the tissue sections from early AMD donors revealed that MPO/NE positive neutrophils (Yellow, white arrows) (**a**) lined the retinal blood vessel

(asterisk) and (**b**) the surface of drusen deposits under the retina (white arrows). (**c**) Control sections showed fewer neutrophils, which did not stain for MPO (asterisk). n=3. Scale bar, 50 μm. Immunofluorescent staining of, (**d-e**) human AMD sections revealed increased staining for citrullinated histone H3 (Green) among neutrophil elastase (NE, Red) positive neutrophils in the retina and choroid (Yellow, white arrows) compared to (**f**) age-matched controls. n=3. Scale bar, 50 μm.

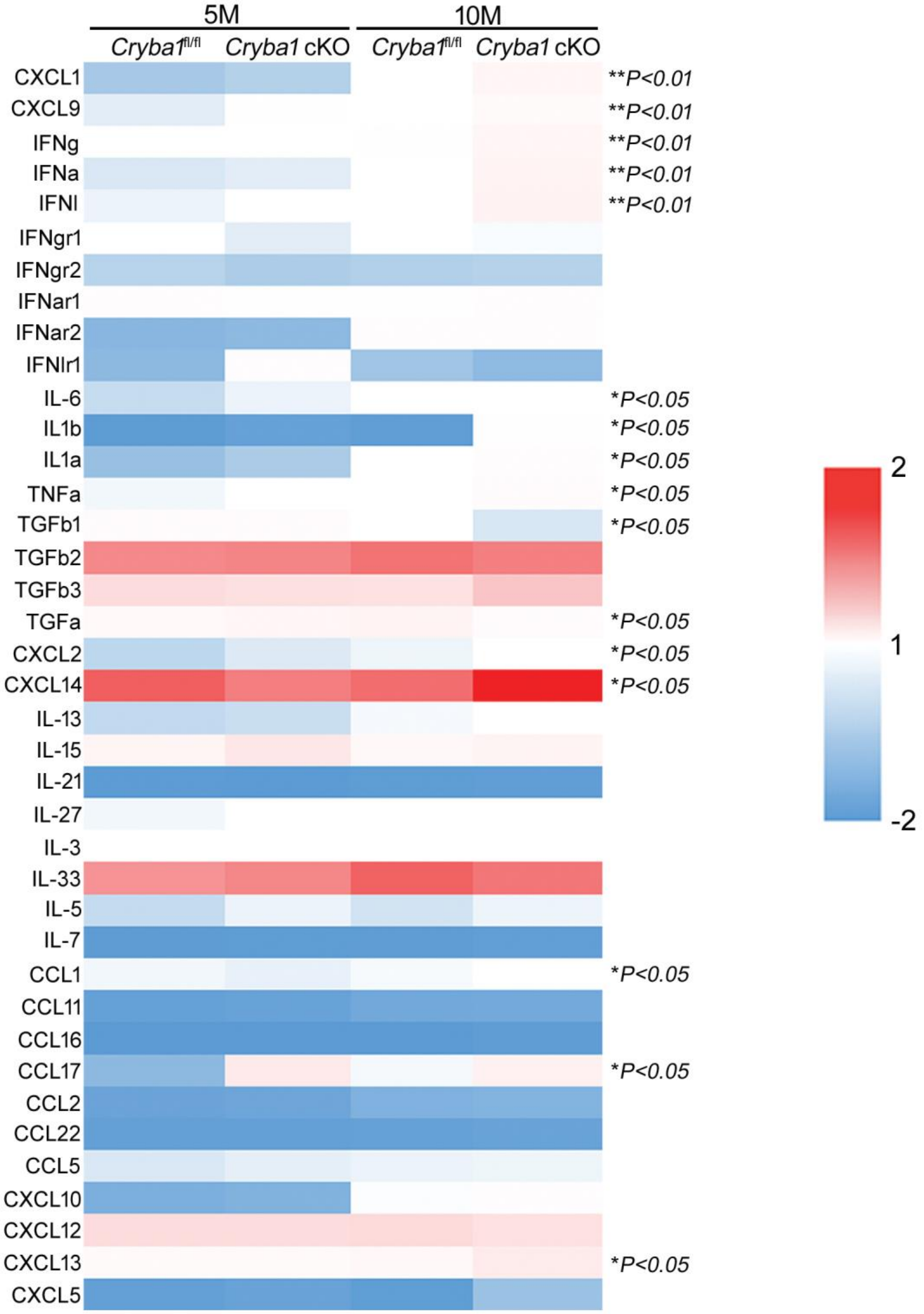

Supplementary Figure 5. Increased transcription of neutrophil-regulating molecules in the RPE/Choroid of mice with AMD-like pathology. Heat map of RNAseq analysis from retina of 5 and 10 month old *Cryba1*$^{fl/fl}$ and *Cryba1* cKO, focusing mainly on the expression of

inflammatory genes. Significant increase in RNA levels of neutrophil regulating molecules like CXCL1, CXCL9 and IFN-family members such as; IFN Type-I (IFNα, IFNβ), Type-II (IFNγ), and IFN Type-III (IFNλ) in retina extracts from 10 month old *Cryba1* cKO mice compared to age-matched *Cryba1*$^{fl/fl}$ (control). No such changes were observed in 5 month old mice, nor were there differences in expression of various IFN receptors. Represents Fragments Per Kilobase of transcript per Million mapped reads (FPKM) for each gene and are represented as log10 (counts per million). n=6. *P< 0.05 and **P< 0.01 with respect to 10 month old *Cryba1*$^{fl/fl}$ group (One-way ANOVA and Tukey's post-hoc test).

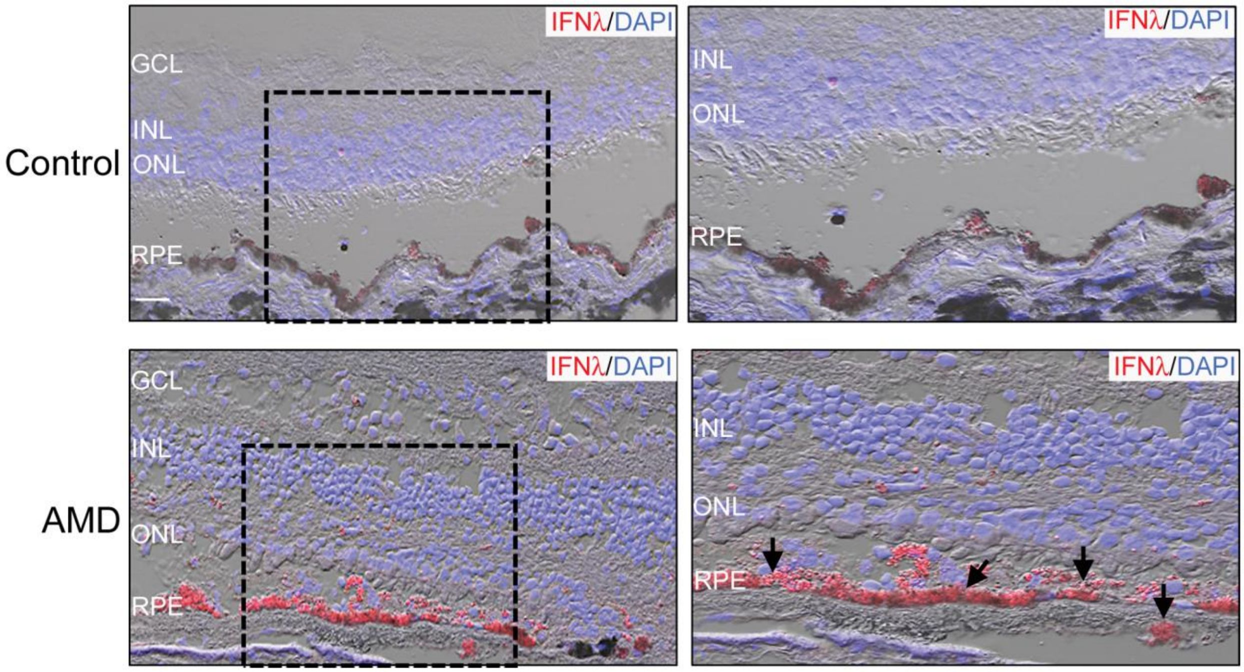

Supplementary Figure 6. IFNλ expression in retina of human AMD patient samples.

Increased IFNλ (Red) immunostaining was apparent in sections from AMD patients relative to age-matched controls. The RPE cells (indicated by arrows) showed increased staining for the protein (zoomed image on right panel representative of ROI-marked with dotted line). The control retina did not show noticeable staining for IFNλ. n=4. Scale Bar, 50 μm.

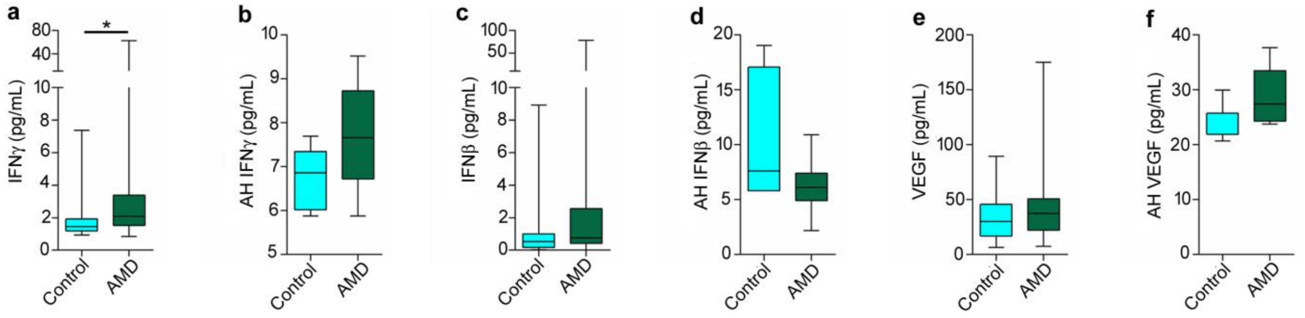

Supplementary Figure 7. Expression of neutrophil regulating cytokines in human samples. Cytometry bead array revealed significant increase in the level of IFNγ in the peripheral blood (PB) in AMD patient samples (**a**) compared to age-matched controls, but not in aqueous humor (AH) samples (**b**). Levels of IFNβ and VEGF did not show any significant change in the PB (**c, e**) or AH (**d,f**) samples between the two groups. Peripheral blood (AMD; n=43 and Controls; n=18) and aqueous humor (AMD; n=6 and Controls; n=7). **P*< 0.05 (Mann-Whitney test). Note: *P*-values for **c-d** are: **c**: 0.09 and **d**: 0.20 (Mann-Whitney test).

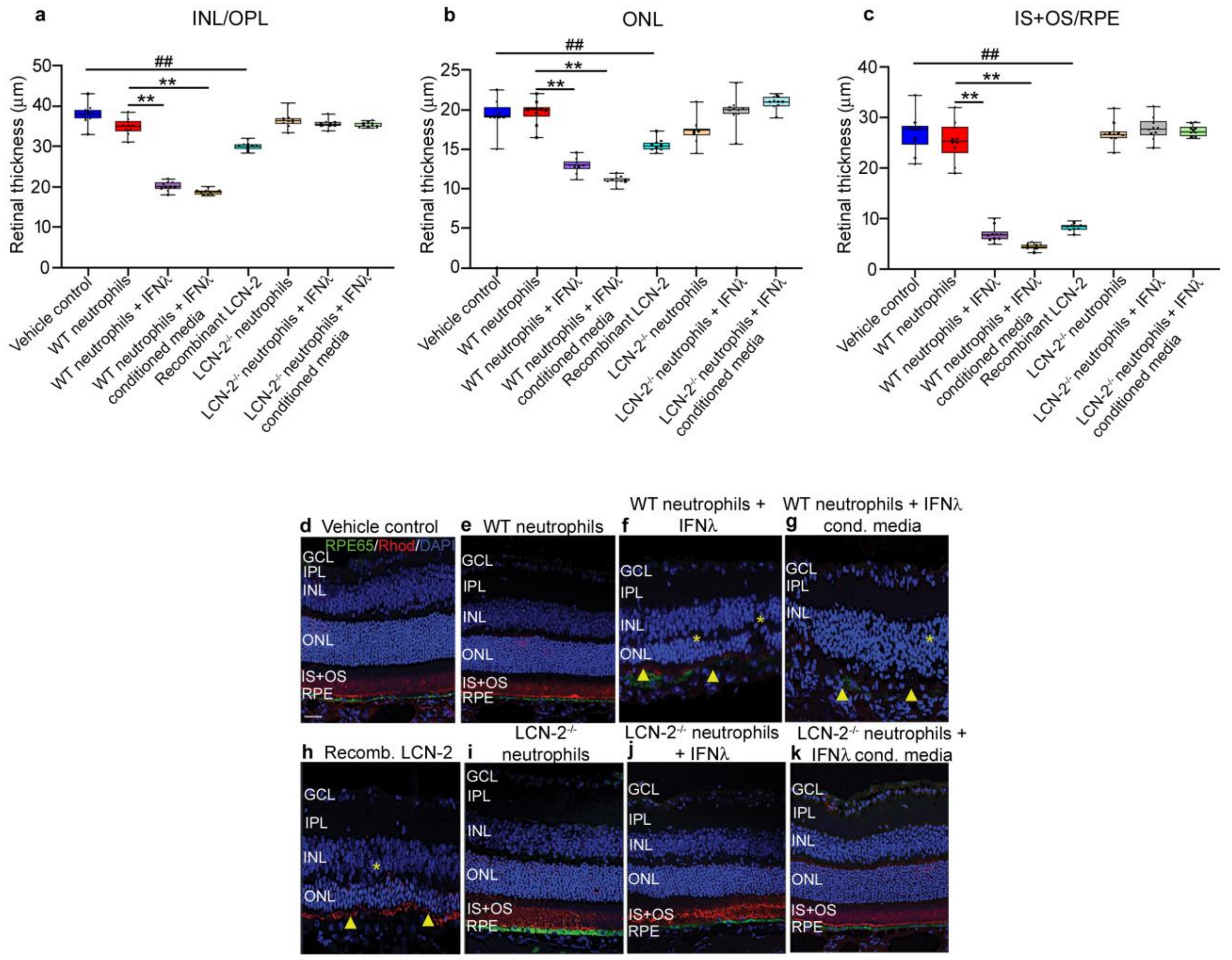

Supplementary Figure 8. Alterations in retinal thickness in NOD-SCID mice injected with LCN- 2 or with neutrophils (WT or LCN-$2^{-/-}$) treated with IFNλ or conditioned medium from RPE cells overexpressing IFNλ. Sub-retinal injections to NOD-SCID mice (Male, 4-5 weeks old) of Recombinant LCN-2 (10 pg/mL) or Wild Type (WT) neutrophils pre-treated with either conditioned media (1:1 diluted) from IFNλ overexpressing RPE cells (6 h) or 200 U/mL recombinant IFNλ for 2 h respectively, demonstrated; decreases in (**a**) INL/OPL, (**b**) ONL and (**c**) IS+OS/RPE thickness compared to vehicle and untreated (control) neutrophil injected groups. No noticeable changes were observed in mice sub-retinally injected with neutrophils from LCN-2 KO mice (LCN-$2^{-/-}$ neutrophils), with or without IFNλ exposure. Thickness (μm) analysis was

performed on optical sections (100 sections per retina) from each eye ranging from -2.0 to +2.0 mm with respect to the optic nerve head (ONH). n=10. ***P*< 0.01 with respect to control neutrophils and ##*P*<0.01 with respect to vehicle control (One-way ANOVA and Tukey's post-hoc test). Immunofluorescence assay on retinas from NOD-SCID mice injected sub-retinally with; (**d**) vehicle or (**f-g**) IFNλ-exposed WT neutrophils or (**h**) recombinant LCN-2 revealed significant loss of IS+OS/RPE layers (yellow arrow heads), evident from decrease in rhodopsin (Red, a marker for rod photoreceptors) and RPE65 (Green, a marker for RPE cells) staining, along with noticeable alterations in the INL/ONL layers (yellow asterisks). Mice injected with (**e**) WT or (**i-k**) LCN-2$^{-/-}$ neutrophils (+/-) IFNλ did not show any change relative to controls. n=5. Scale Bar, 50 μm.

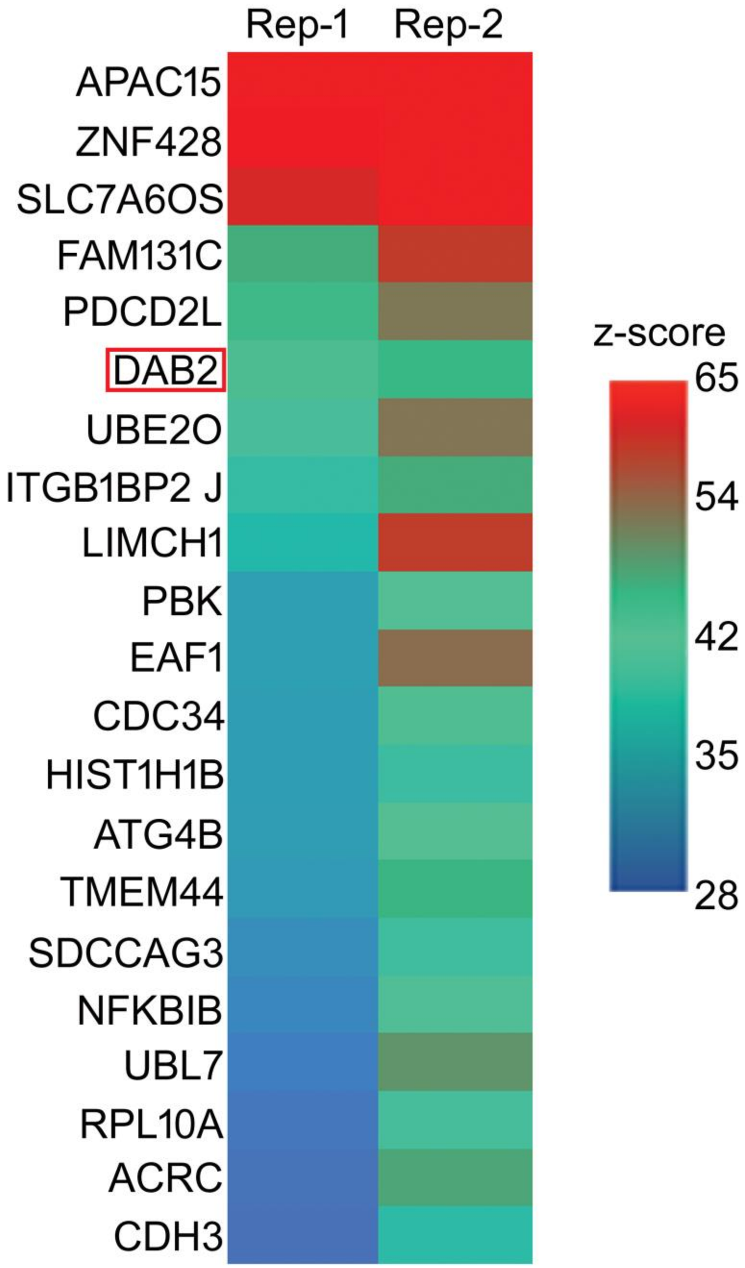

Supplementary Figure 9. DAB2 interacts with LCN-2. Human proteome array showing binding partners of LCN-2 including DAB2 (red box) probed on HuProtTM arrays at 1 μg/ml. Represented as z-score (hit for each probe), with a cut-off of 6 and values ranging from 28 to 65. n=3.

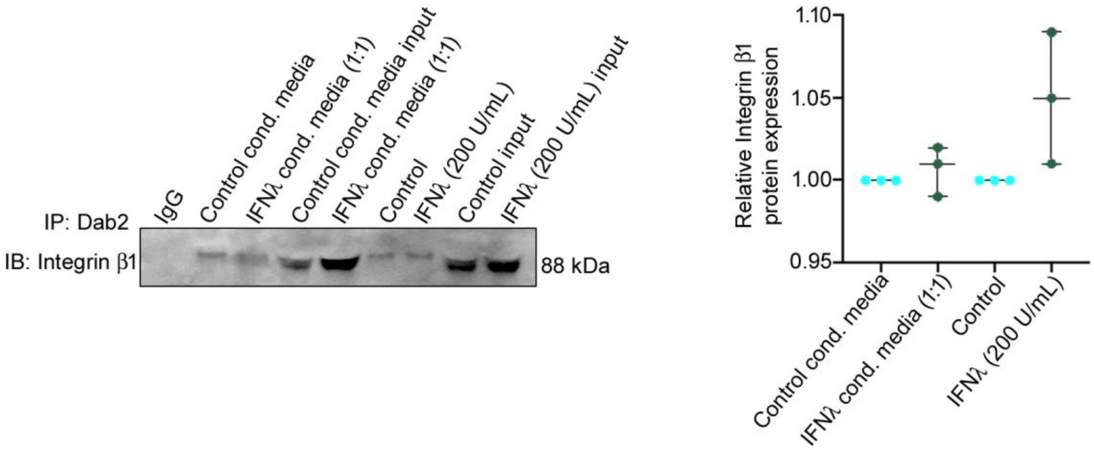

Supplementary Figure 10. Dab2 binds to integrin β1. Pull down assay from immunoprecipitated Dab2 was used to determine the expression of integrin β1 by western analysis from wild type neutrophils treated with either recombinant IFNλ (200 U/mL) or IFNλ conditioned media (1:1). This revealed that Dab2 binds to integrin β1 and there is no noticeable change in the binding pattern between the two proteins upon IFNλ exposure. n=3.

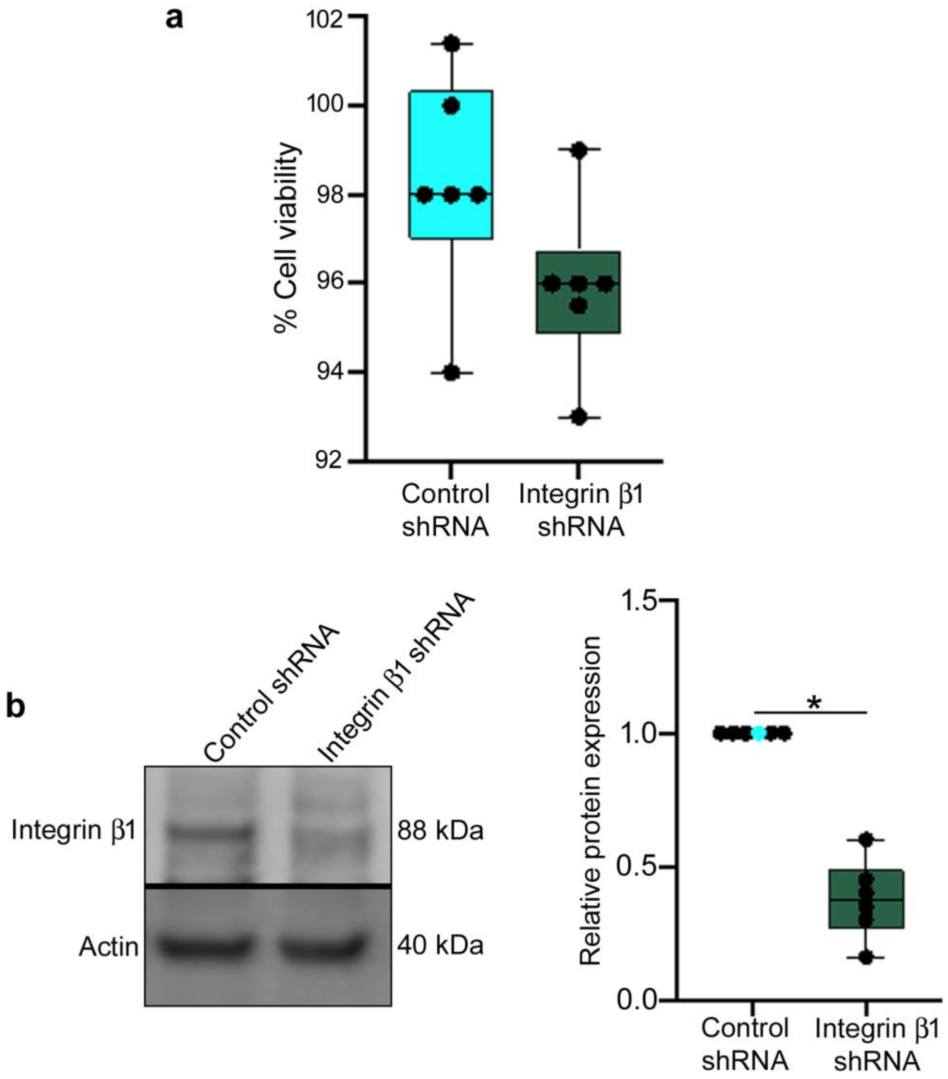

Supplementary Figure 11. Inhibition of integrin β1 expression in neutrophils. Wild type neutrophils in culture were transfected with integrin β1 shRNA viral particles (see methods). (**a**) MTT (3-(4,5-Dimethylthiazol-2-Yl)-2,5-Diphenyltetrazolium Bromide) cell viability assay revealed no significant change in % cell viability between control shRNA and integrin β1 shRNA transfected neutrophils. n=6. (**b**) Decreased expression of integrin β1 as evident from immunoblot and densitometry among integrin β1 shRNA-transfected neutrophils, relative to control shRNA transfected cells. n=6. *$P < 0.05$ (one-way ANOVA and Tukey's post-hoc test).

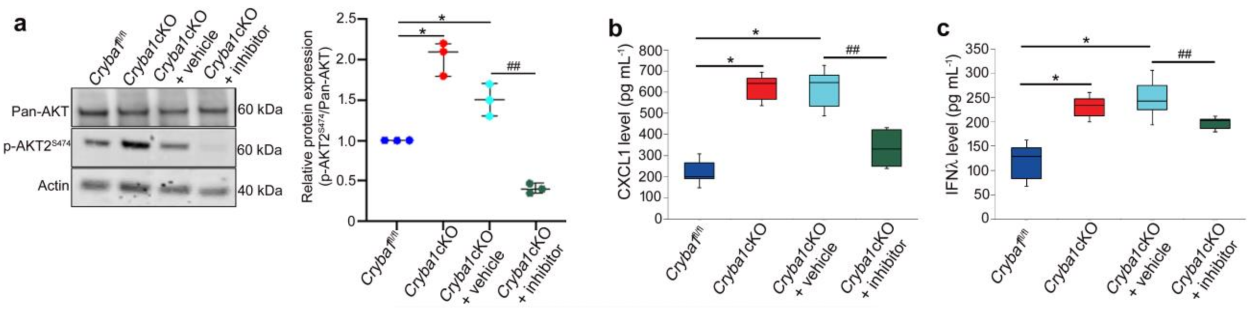

Supplementary Figure 12. An AKT2 inhibitor (CCT128930) reduces inflammation in aged *Cryba1* cKO mouse retina. (**a**) Immunoblot and summary of densitometry showing a significant increase in the phosphorylation of AKT2 (p-AKT2^{S474}) in retinas from 1 year old *Cryba1* cKO mice. The levels of pAKT2^{S474} in the *Cryba1* cKO RPE decreased significantly following treatment with inhibitor (CCT128930, at a dose of 500 μM). Vehicle alone (2.5% DMSO in PBS) had little effect. Additionally, levels of total AKT did not change in the samples. n=3. *P< 0.05 with respect to floxed control and $^{\#\#}P$< 0.01 with respect to vehicle treated *Cryba1* cKO. (**b-c**) ELISA assays show reduced levels (pg/mL) of CXCL1 and IFNλ respectively, in the RPE-choroid of AKT2 inhibitor-treated *Cryba1* cKO mice, as compared to age-matched vehicle and untreated *Cryba1* cKO animals. n=3. *P< 0.05 with respect to floxed control and $^{\#\#}P$< 0.01 with respect to vehicle treated *Cryba1* cKO.

Supplementary Figure 13. Full uncropped blot/gel images.

Figure 1B

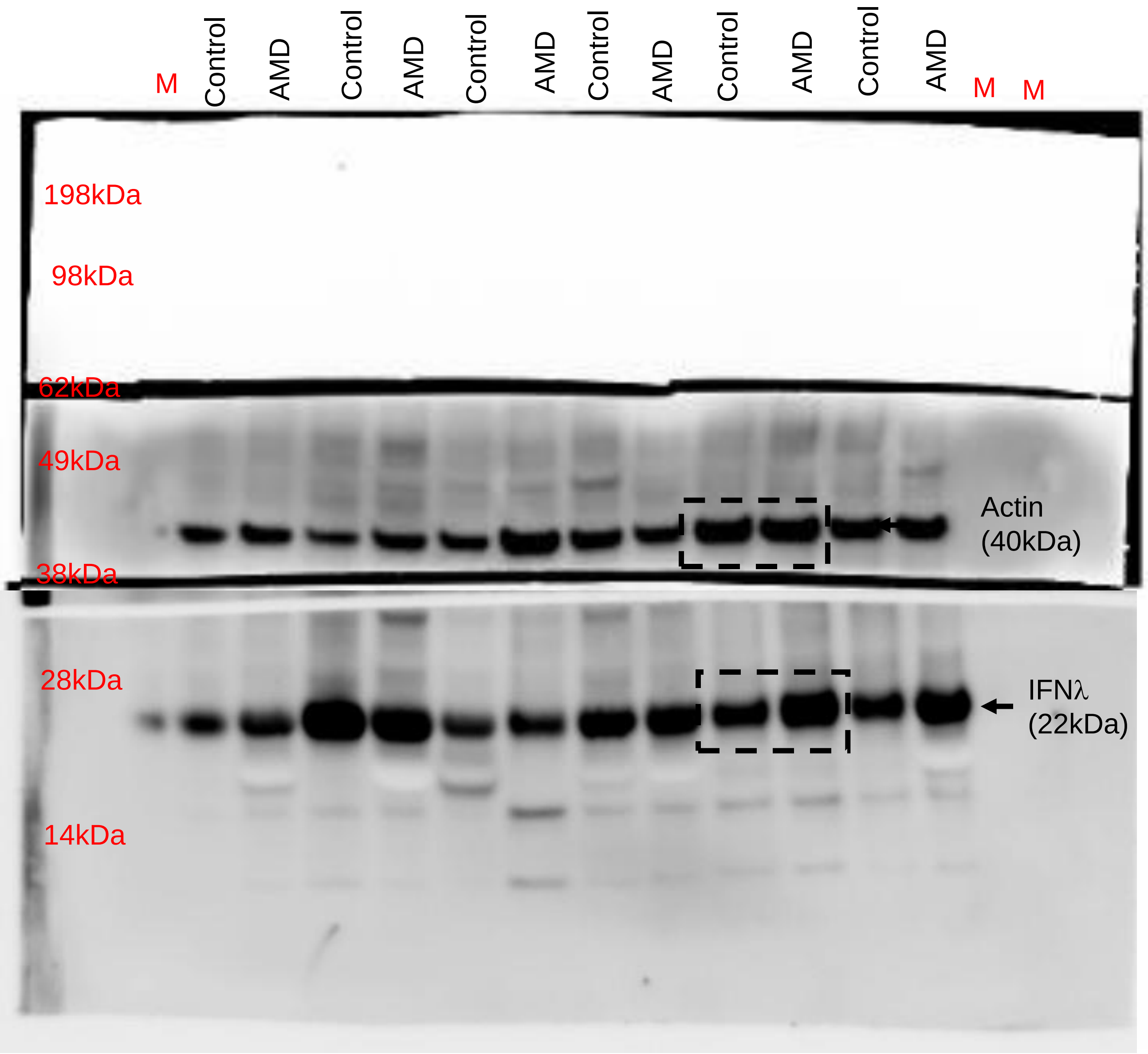
M
Control
AMD
Control
AMD
Control
AMD
Control
AMD
Control
AMD
Control
AMD
M
M
198kDa
98kDa
62kDa
49kDa
38kDa
28kDa
14kDa
Actin
(40kDa)
IFNλ
(22kDa)

Figure 1B

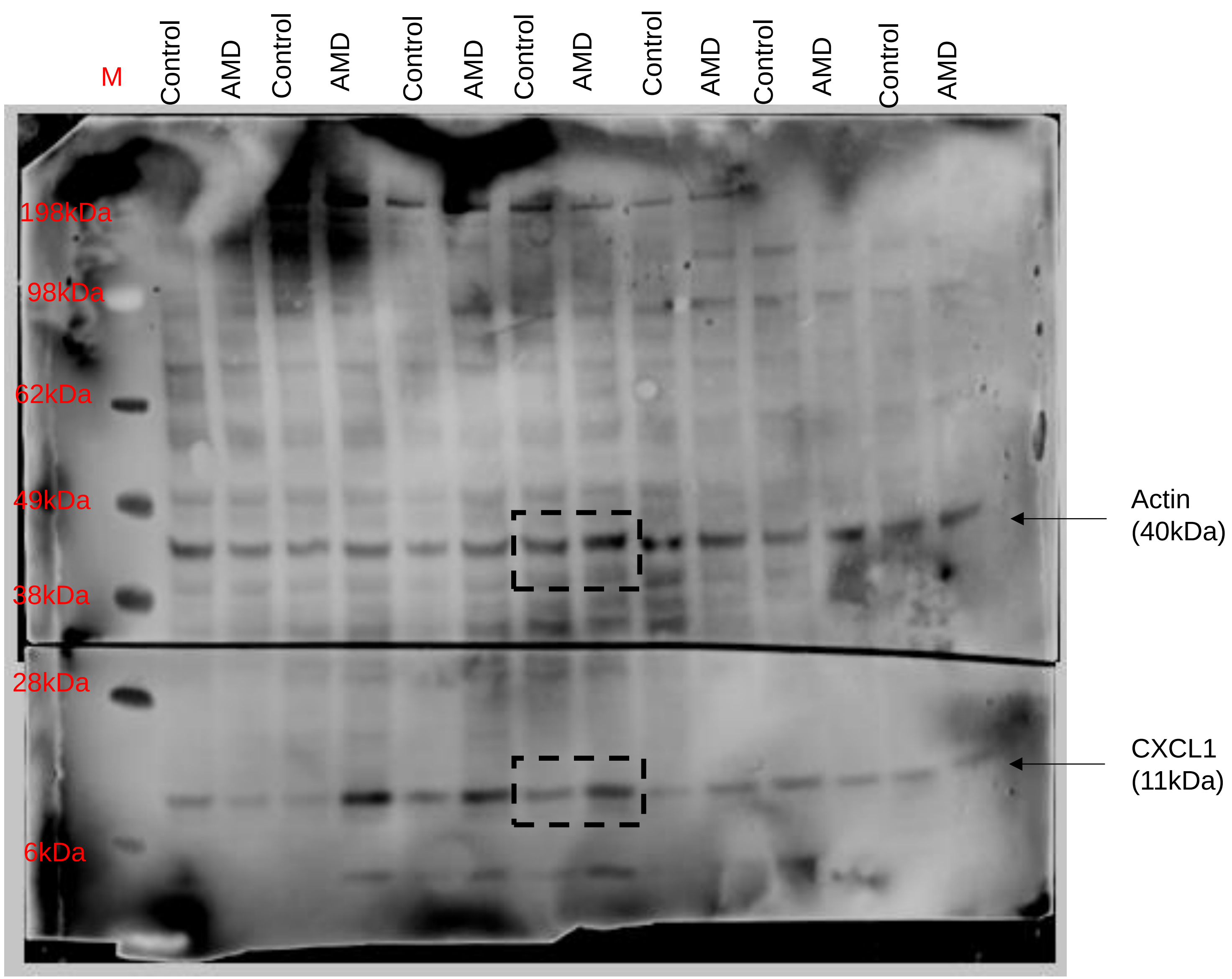
M
Control
AMD
Control
AMD
Control
AMD
Control
AMD
Control
AMD
Control
AMD
Control
AMD
198kDa
98kDa
62kDa
49kDa
38kDa
28kDa
6kDa
Actin
(40kDa)
CXCL1
(11kDa)

Figure 3A

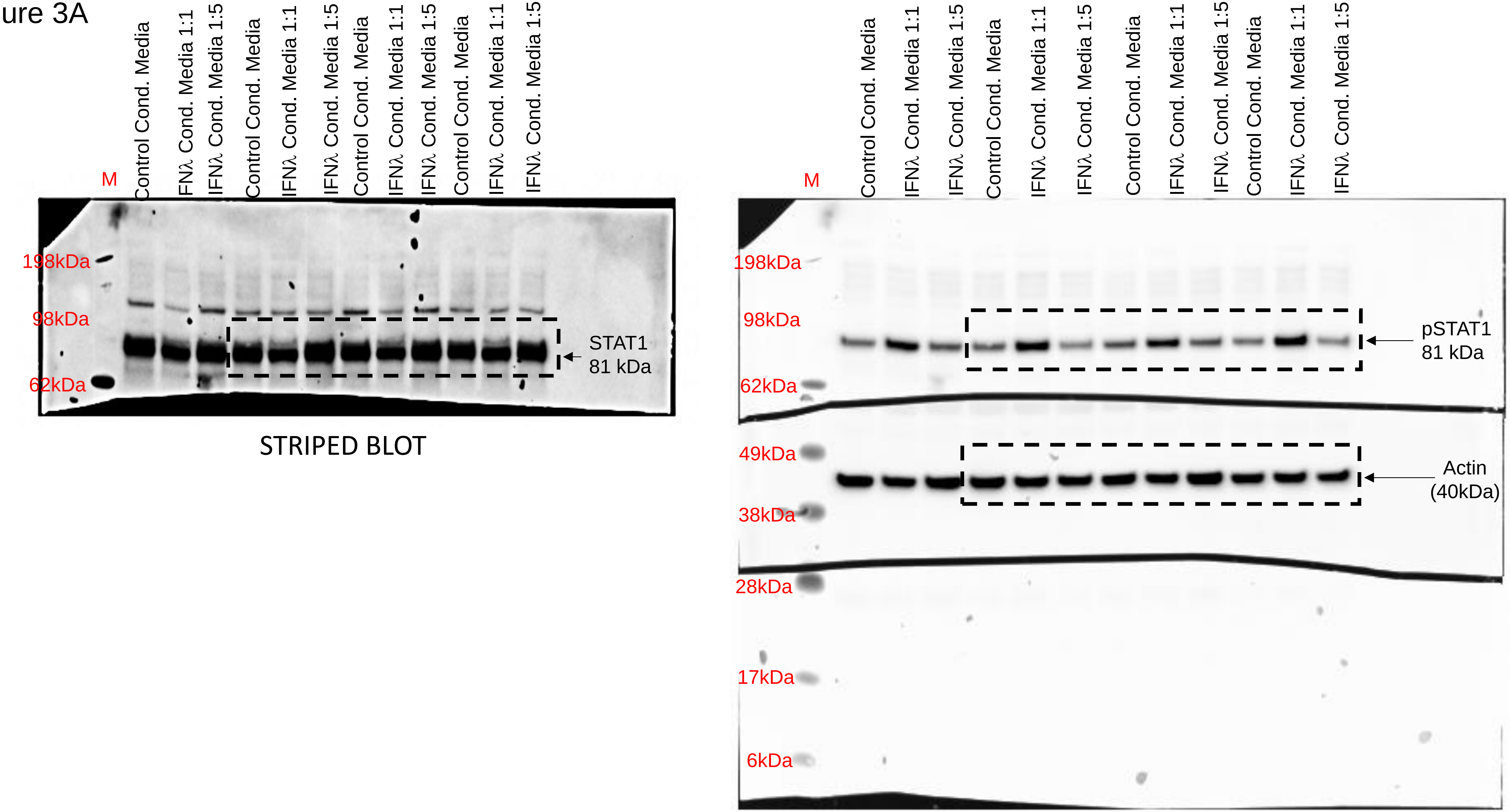

M
Control Cond. Media
IFNλ Cond. Media 1:1
IFNλ Cond. Media 1:5
Control Cond. Media
IFNλ Cond. Media 1:1
IFNλ Cond. Media 1:5
Control Cond. Media
IFNλ Cond. Media 1:1
IFNλ Cond. Media 1:5
Control Cond. Media
IFNλ Cond. Media 1:1
IFNλ Cond. Media 1:5
198kDa
98kDa
62kDa
STAT1
81 kDa
STRIPED BLOT
M
198kDa
98kDa
62kDa
49kDa
38kDa
28kDa
17kDa
6kDa
pSTAT1
81 kDa
Actin
(40kDa)

Figure 3A

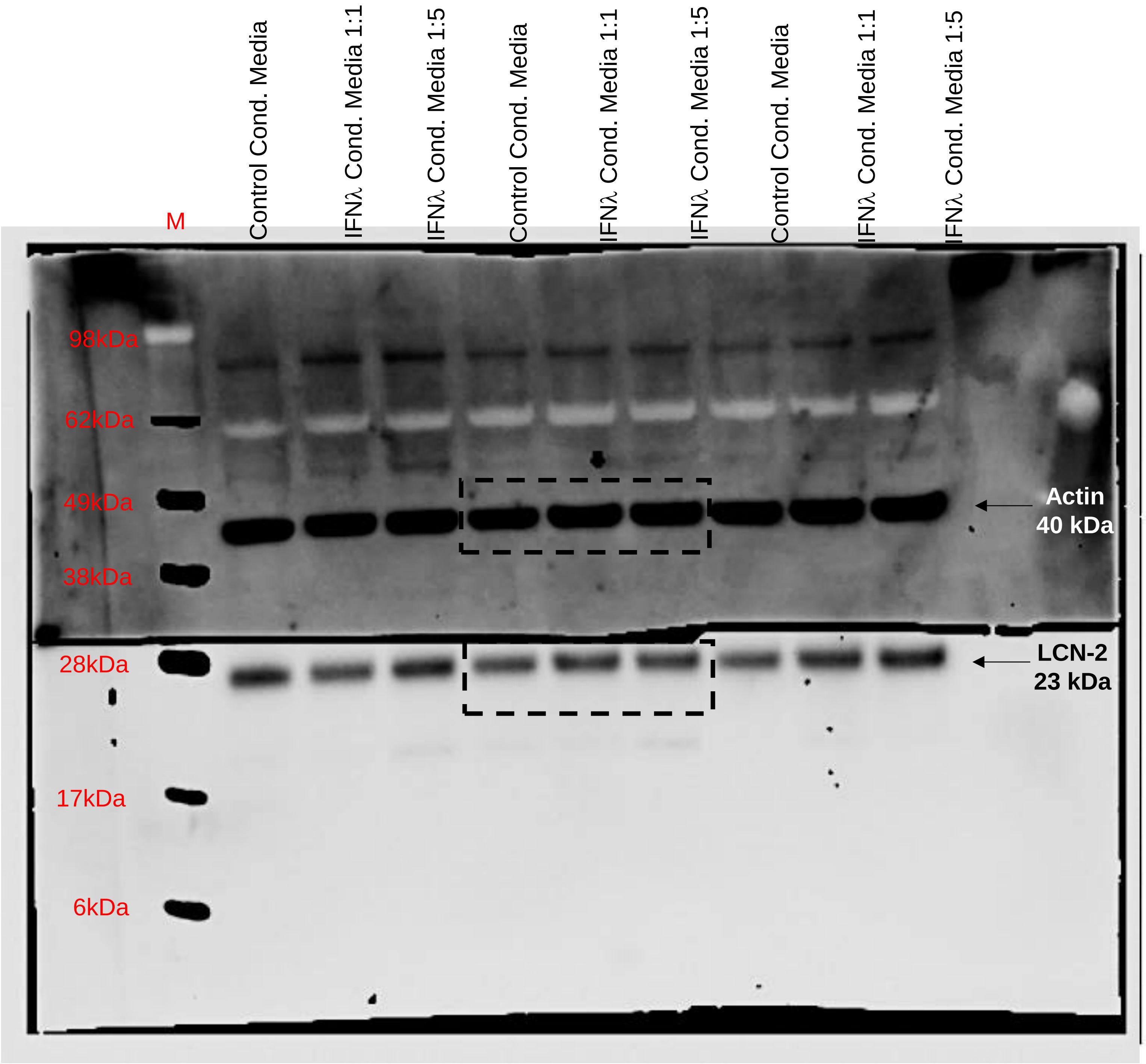

M
Control Cond. Media
IFNλ Cond. Media 1:1
IFNλ Cond. Media 1:5
Control Cond. Media
IFNλ Cond. Media 1:1
IFNλ Cond. Media 1:5
Control Cond. Media
IFNλ Cond. Media 1:1
IFNλ Cond. Media 1:5
98kDa
62kDa
49kDa
38kDa
28kDa
17kDa
6kDa
Actin
40 kDa
LCN-2
23 kDa

Figure 3A

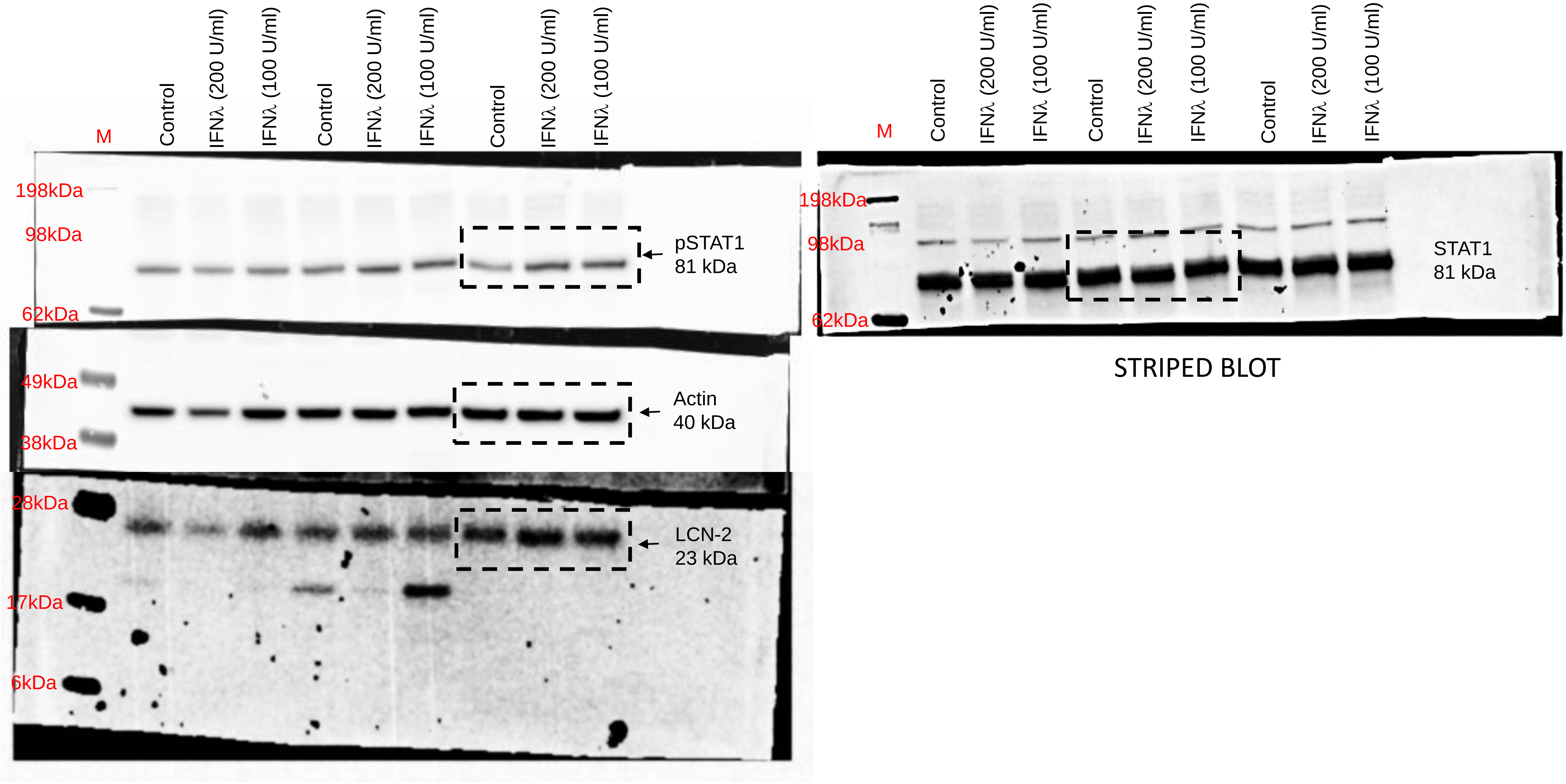

M
Control
IFNλ (200 U/ml)
IFNλ (100 U/ml)
Control
IFNλ (200 U/ml)
IFNλ (100 U/ml)
Control
IFNλ (200 U/ml)
IFNλ (100 U/ml)
198kDa
98kDa
62kDa
pSTAT1
81 kDa
49kDa
38kDa
Actin
40 kDa
28kDa
17kDa
6kDa
LCN-2
23 kDa
M
Control
IFNλ (200 U/ml)
IFNλ (100 U/ml)
Control
IFNλ (200 U/ml)
IFNλ (100 U/ml)
Control
IFNλ (200 U/ml)
IFNλ (100 U/ml)
198kDa
98kDa
62kDa
STAT1
81 kDa
STRIPED BLOT

Figure 7A

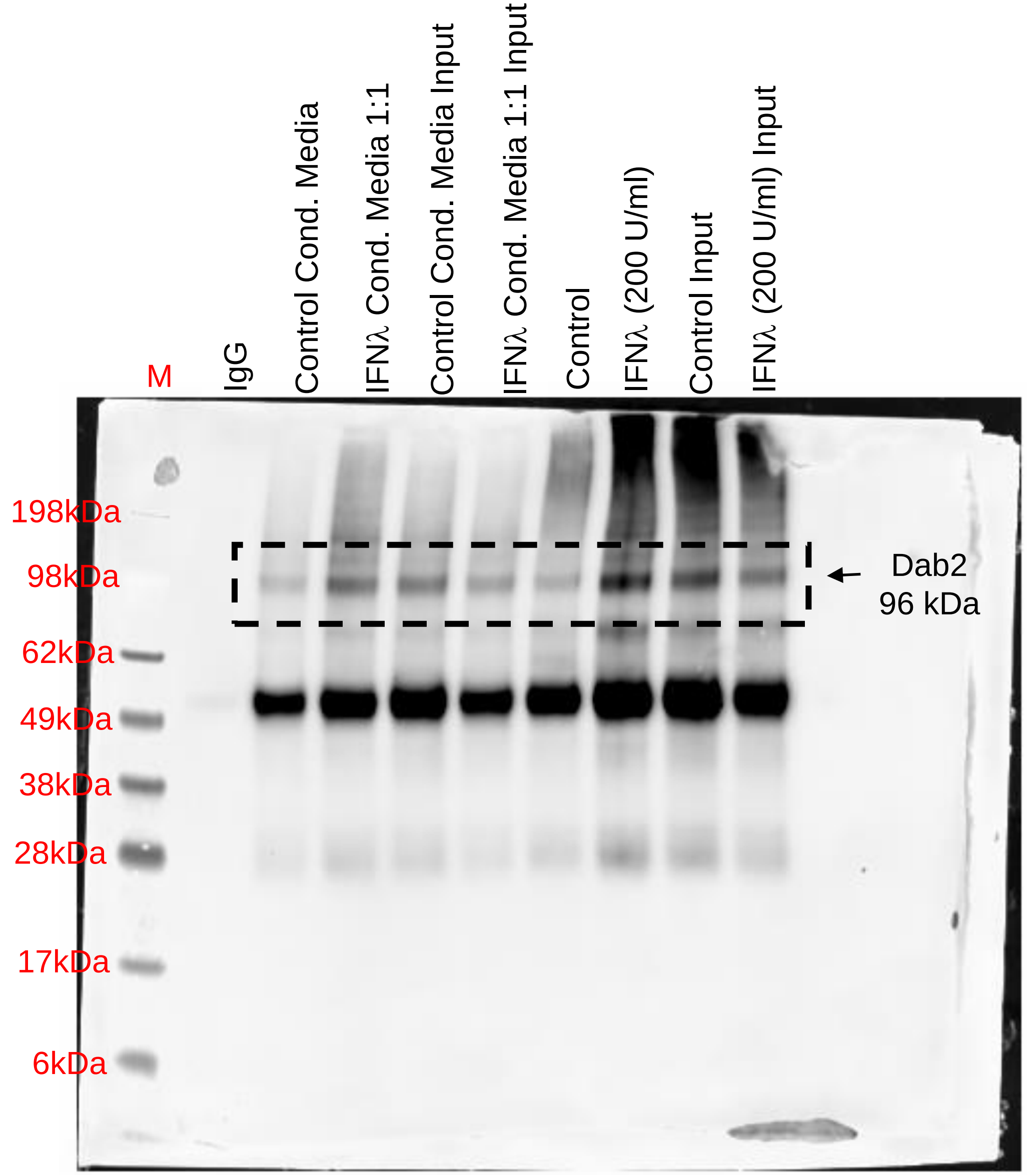
M
IgG
Control Cond. Media
IFNλ Cond. Media 1:1
Control Cond. Media Input
IFNλ Cond. Media 1:1 Input
Control
IFNλ (200 U/ml)
Control Input
IFNλ (200 U/ml) Input
198kDa
98kDa
62kDa
49kDa
38kDa
28kDa
17kDa
6kDa
Dab2
96 kDa

Figure S10

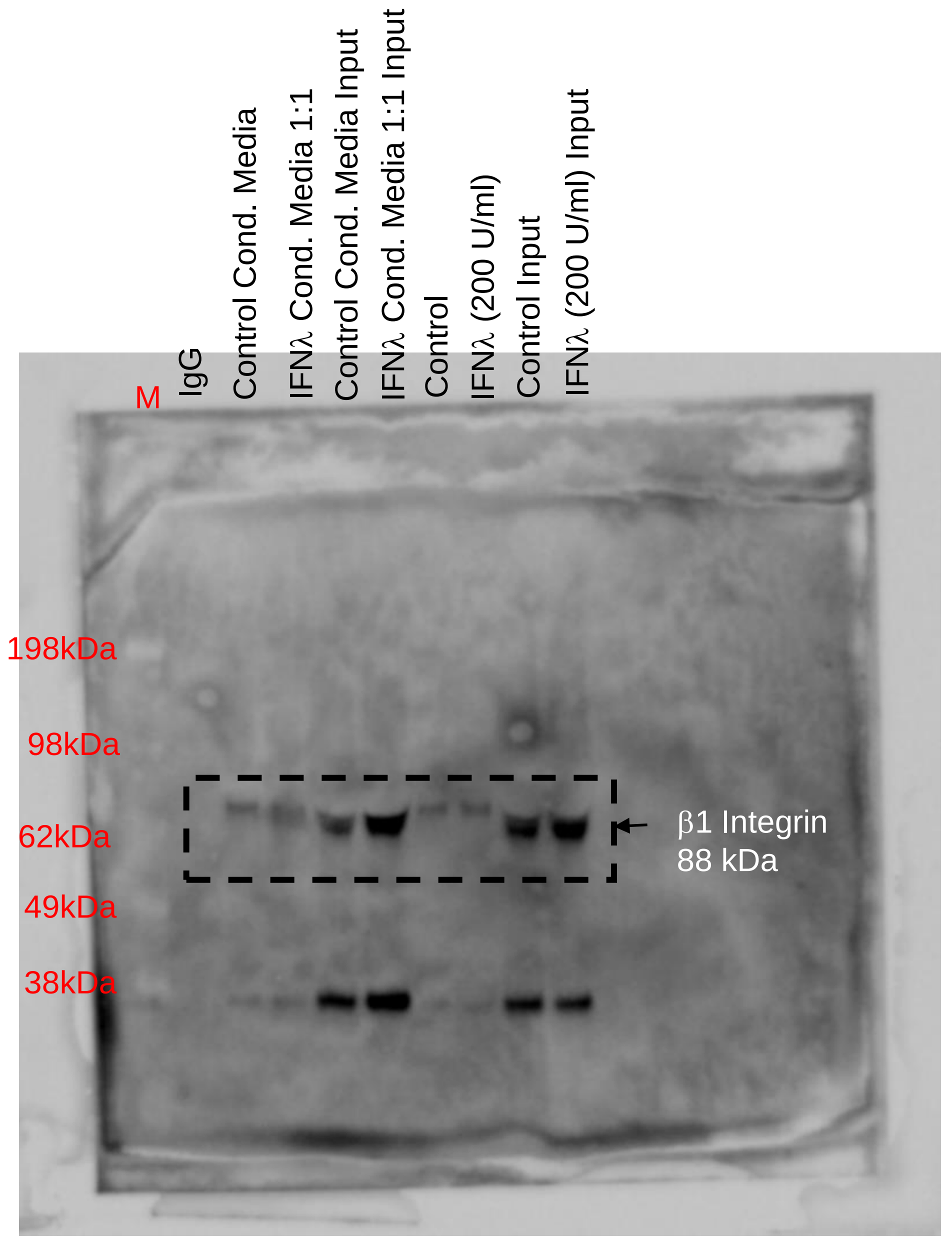
M
IgG
Control Cond. Media
IFNλ Cond. Media 1:1
Control Cond. Media Input
IFNλ Cond. Media 1:1 Input
Control
IFNλ (200 U/ml)
Control Input
IFNλ (200 U/ml) Input
198kDa
98kDa
62kDa
49kDa
38kDa
β1 Integrin
88 kDa

Figure S11B

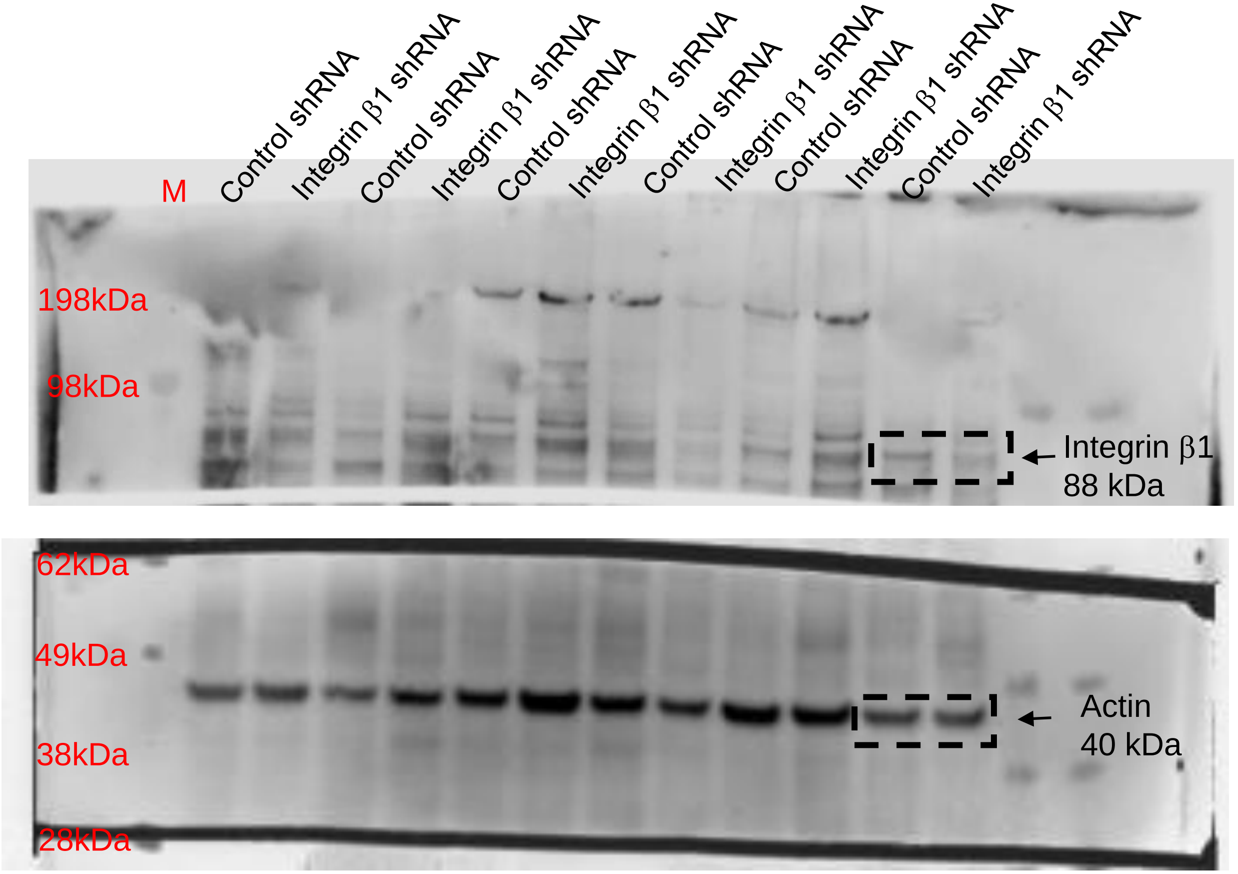

M
Control shRNA
Integrin β1 shRNA
Control shRNA
Integrin β1 shRNA
Control shRNA
Integrin β1 shRNA
Control shRNA
Integrin β1 shRNA
Control shRNA
Integrin β1 shRNA
Control shRNA
Integrin β1 shRNA
198kDa
98kDa
Integrin β1
88 kDa
62kDa
49kDa
38kDa
28kDa
Actin
40 kDa

Figure S12 A

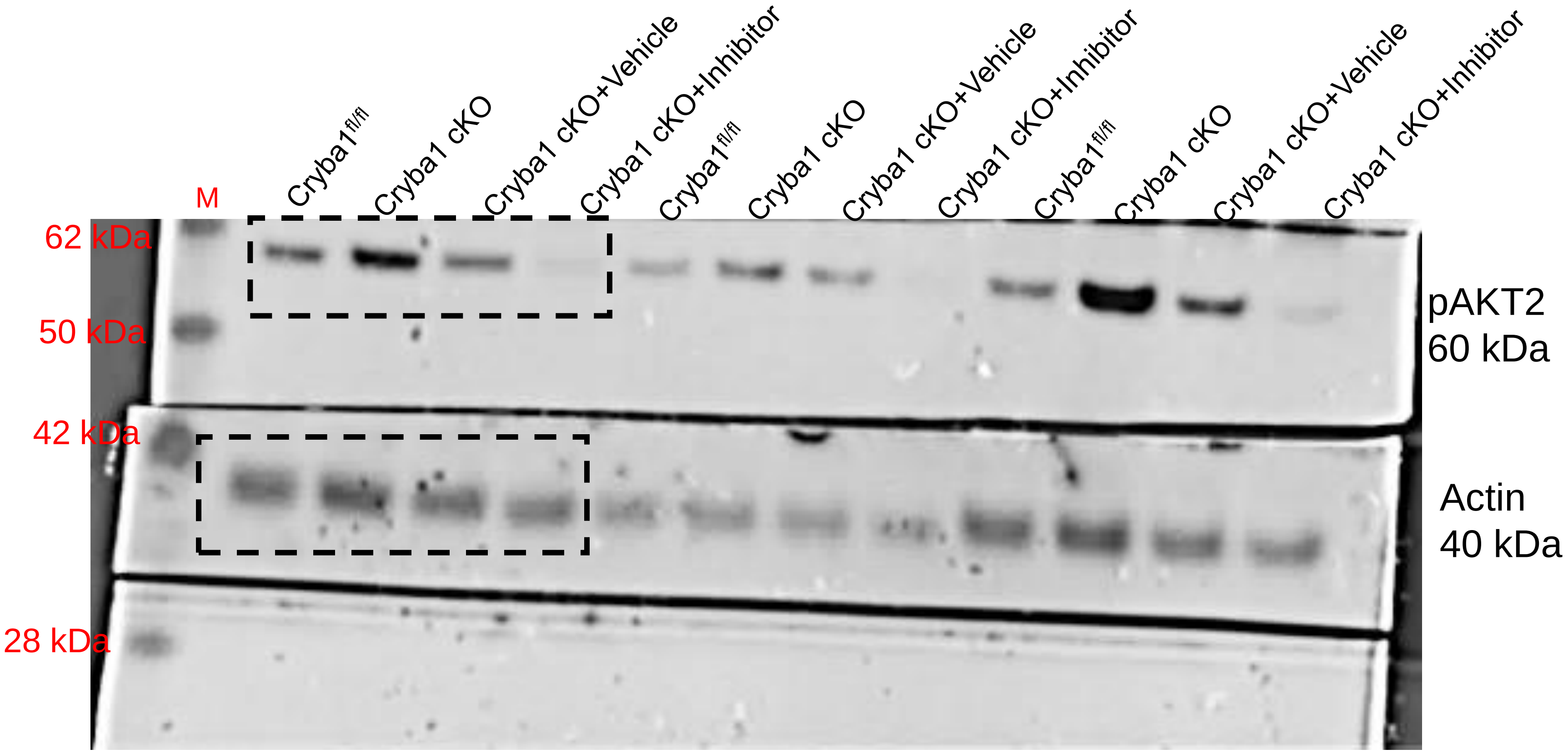
M
Cryba1fl/fl
Cryba1 cKO
Cryba1 cKO+Vehicle
Cryba1 cKO+Inhibitor
Cryba1fl/fl
Cryba1 cKO
Cryba1 cKO+Vehicle
Cryba1 cKO+Inhibitor
Cryba1fl/fl
Cryba1 cKO
Cryba1 cKO+Vehicle
Cryba1 cKO+Inhibitor
62 kDa
50 kDa
42 kDa
28 kDa
pAKT2
60 kDa
Actin
40 kDa

Figure S12 A

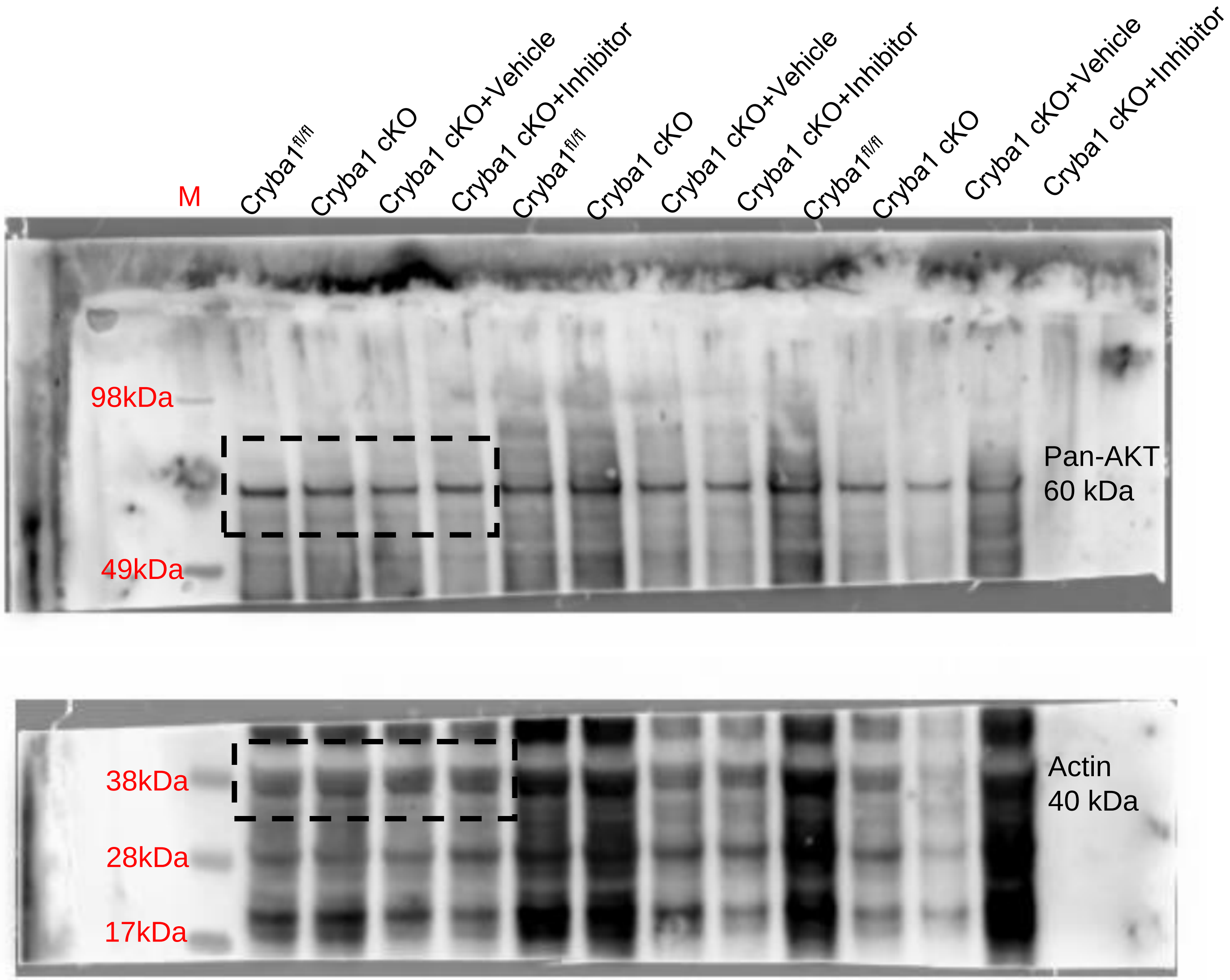
M
Cryba1fl/fl
Cryba1 cKO
Cryba1 cKO+Vehicle
Cryba1 cKO+Inhibitor
Cryba1fl/fl
Cryba1 cKO
Cryba1 cKO+Vehicle
Cryba1 cKO+Inhibitor
Cryba1fl/fl
Cryba1 cKO
Cryba1 cKO+Vehicle
Cryba1 cKO+Inhibitor
98kDa
49kDa
Pan-AKT
60 kDa
38kDa
28kDa
17kDa
Actin
40 kDa

a

	Control (n=18)	AMD (n=43)	*P*-value
Age (Mean±SEM; Range) Years	61.3±14.4; 43-77	68.1±10.4;51-88	0.016
Gender (M/F)	10/8	23/20	NA
Log Mar (BCVA) RE	0.08±0.02; 0-0.30	0.32±0.05;0-1.61	0.010
Log Mar (BCVA) LE	0.14±0.03; 0-0.78	0.20±0.03;0-0.78	0.276

b

	Control (n=7)	AMD (n=6)	*P*-value
Age (Mean±SEM; Range) Years	60.4±3.2;53-76	63±3.5;55-76	0.462
Gender (M/F)	3/4	3/3	NA
Log Mar (BCVA)	0.53±0.3;0.1-2.1	0.23±0.06;0.03-0.5	0.463

Supplementary Table 1. Cohort characteristics of control subjects and AMD patients , (**a**) Cohort details of subjects included for immunophenotyping and soluble factors quantification in peripheral blood and plasma, respectively (**b**) Cohort details of subjects included for soluble factors quantification in aqueous humor,
