# Supplementary material for: Neutrophils homing into the retina trigger pathology in early age-related macular degeneration
Source: Commun Biol. 2019 Sep 20;2:348. doi: 10.1038/s42003-019-0588-y (PMC6754381; doi:10.1038/s42003-019-0588-y)
Supplement: Supplementary file 2 — Description of Additional Supplementary Files [file 42003_2019_588_MOESM2_ESM.docx]

**Description of Additional Supplementary Files**

**File Name**: Supplementary Movie 1

**Description**: MP4 file, RSCM image acquisition along with 3-Dimensional rendering of gross whole eye morphology and cross-sectional image acquisition showing infiltrating red CMTPX-tagged neutrophils in the retina and Schlemm's canal (a channel at the limbus, which is the joining point of the cornea and sclera, encircling the cornea) among intravenously injected NOD-SCID mice treated with IFNλ-exposed WT neutrophils.

**File Name:** Supplementary Data 1

**Description:** Supplementary Figure 14. Source data from main figure graphs uploaded separately as an Excel file.
