# Supplementary material for: Neutrophils homing into the retina trigger pathology in early age-related macular degeneration
Source: Commun Biol. 2019 Sep 20;2:348. doi: 10.1038/s42003-019-0588-y (PMC6754381; doi:10.1038/s42003-019-0588-y)
Supplement: Supplementary file 5 — Reporting Summary [file 42003_2019_588_MOESM5_ESM.pdf]

natureresearch

Corresponding author(s): Debasish Sinha

Last updated by author(s): Aug 23, 2019

Reporting Summary

Nature Research wishes to improve the reproducibility of the work that we publish. This form provides structure for consistency and transparency in reporting. For further information on Nature Research policies, seeAuthors & Referees and theEditorial Policy Checklist .

Statistics

For all statistical analyses, confirm that the following items are present in the figure legend, table legend, main text, or Methods section.

n/a	Confirmed	
☐	☒	The exact sample size (*n*) for each experimental group/condition, given as a discrete number and unit of measurement
☐	☒	A statement on whether measurements were taken from distinct samples or whether the same sample was measured repeatedly
☐	☒	The statistical test(s) used AND whether they are one- or two-sided<br>*Only common tests should be described solely by name; describe more complex techniques in the Methods section.*
☐	☒	A description of all covariates tested
☐	☒	A description of any assumptions or corrections, such as tests of normality and adjustment for multiple comparisons
☐	☒	A full description of the statistical parameters including central tendency (e.g. means) or other basic estimates (e.g. regression coefficient) AND variation (e.g. standard deviation) or associated estimates of uncertainty (e.g. confidence intervals)
☐	☒	For null hypothesis testing, the test statistic (e.g. *F*, *t*, *r*) with confidence intervals, effect sizes, degrees of freedom and *P* value noted<br>*Give P values as exact values whenever suitable.*
☒	☐	For Bayesian analysis, information on the choice of priors and Markov chain Monte Carlo settings
☒	☐	For hierarchical and complex designs, identification of the appropriate level for tests and full reporting of outcomes
☒	☐	Estimates of effect sizes (e.g. Cohen's *d*, Pearson's *r*), indicating how they were calculated

Our web collection on statistics for biologists contains articles on many of the points above.

Software and code

Policy information about availability of computer code

Data collection	FACSDIVA software, Diver 2.4 software (Bioptigen), MATLAB v2017b, Imaris 9.2.1 (Bitplane)
Data analysis	Microsoft Excel, Graphpad prism 6, FlowJo (version 7.6.5), ImageJ software

For manuscripts utilizing custom algorithms or software that are central to the research but not yet described in published literature, software must be made available to editors/reviewers. We strongly encourage code deposition in a community repository (e.g. GitHub). See the Nature Research guidelines for submitting code & software for further information.

Data

Policy information about availability of data

All manuscripts must include a data availability statement. This statement should provide the following information, where applicable:

- Accession codes, unique identifiers, or web links for publicly available datasets
- A list of figures that have associated raw data
- A description of any restrictions on data availability

All data generated or analysed during this study are included in this published article (and its Supplementary Information files). The raw uncropped western blot images are supplied in a single file as Supplementary Figure 13. The source data underlying the graphs and charts presented in the main figures are available as Supplementary Figure 14. The RNAseq data for the genes depicted in Supplementary Figure 5 are uploaded in the NCBI GEO database (Accession Number: GSE136280).

Field-specific reporting

Please select the one below that is the best fit for your research. If you are not sure, read the appropriate sections before making your selection.

Life sciences study design

All studies must disclose on these points even when the disclosure is negative.

Sample size	Sample sizes was determined based on power calculation, showing a difference of 25% and up to 20% SD, alpha=0.05 and beta=0.2.
Data exclusions	No data were excluded, but some animals (<1%) were excluded from the experiments due to technical challenges in the procedures such as animal death due to anesthesia complications.
Replication	Repeated experiments (atleast three repeats) were performed to reliably reproduce the results of the experiments.
Randomization	Animals (from respective genotypes) were allocated to each groups (seperated in cages) randomly.
Blinding	To eliminate biasness, individuals handling core-facility instruments or performing analysis on specific experiments were blinded to mouse genotype identity as well as the identity of the experimental groups.

Reporting for specific materials, systems and methods

We require information from authors about some types of materials, experimental systems and methods used in many studies. Here, indicate whether each material, system or method listed is relevant to your study. If you are not sure if a list item applies to your research, read the appropriate section before selecting a response.

Materials & experimental systems

n/a	Involved in the study
☐	☒ Antibodies
☒	☐ Eukaryotic cell lines
☒	☐ Palaeontology
☐	☒ Animals and other organisms
☐	☒ Human research participants
☒	☐ Clinical data

Methods

n/a	Involved in the study
☒	☐ ChIP-seq
☐	☒ Flow cytometry
☒	☐ MRI-based neuroimaging

Antibodies

Antibodies used	PE/Cy7-tagged CD45 (Cat# 103114), APC-tagged Ly6C (Cat# 128016), FITC-tagged CD66b (Cat# 555724), V450-tagged Ly6G (Cat# 650603), Alexa fluor 700-tagged CD11b (Cat# 557960), anti-human PE/Cy7-tagged CD45 (Cat# 560178), anti-human CD34 antibody (Cat# 343602) were purchased from BD Biosciences, USA and anti-human PE-tagged IL-28AR antibody (Cat# 337804) was purchased from Biolegend, USA. Anti-Neutrophil Elastase (Cat# ab68672), anti-GRO alpha (CXCL1) (Cat# ab86436), anti-STAT1 (phospho S727) (Cat# ab109461), anti-IL-28 receptor alpha or IL28R1 (Cat# ab224395), anti-Histone H3 citrunillated (Cat# ab219407), VCAM1 (Cat# ab134047), CD34 (Cat# 8158) and IL28+ IL29 (Cat# ab191426) antibodies were purchased from Abcam, USA. Anti-ICAM-1 (Cat# SC-107), anti-STAT1 (Cat# 9172T), anti-AKT (Cat# 4685S), anti-AKT2 (Cat# 2964S) and anti-DAB2 (Cat# 12906S) were purchased from Cell Signaling Technologies, USA. Other antibodies used include: Alexa-fluor 488-tagged beta1 integrin (Santa Cruz Biotechnology, USA; Cat# sc-374429 AF488), anti-IL-28A/IFNlambda2 (Antibodies online, USA; Cat# ABIN357173), anti-Ly6G (Antibodies online, USA; Cat# ABIN1854937), IL-29 antibody (Biorbyt, USA; Cat# orb6201), anti-IFNalpha (Thermo Fisher, USA; Cat# 221001), anti-Myeloperoxidase/MPO (R&D Systems, USA; Cat# AF3667-SP), anti-LCN-2 (EMD Millipore; Cat# AB2267) and anti-Actin (Sigma-Aldrich, USA; Cat# A2066). Primary antibodies were used at a dilution of 1:1000 for western blotting and 1:100 for immunohistochemistry. Secondary antibodies were used at a dilution of 1:2500 for western blotting and 1:300 for immunohistochemistry.
Validation	The validation information for each antibody is cited in the methods section with catalogue numbers provided by the manufacturers.

Animals and other organisms

Policy information about studies involving animals; ARRIVE guidelines recommended for reporting animal research

Laboratory animals	Male mice (C57BL/6J background) and NOD-SCID mice (Male) were used in the study. Additional detailed information regarding the genotype and age is provided in methods section.
Wild animals	N/A
Field-collected samples	N/A
Ethics oversight	All animal studies were conducted in accordance with the Guide for the Care and Use of Animals (National Academy Press) and were approved by the University of Pittsburgh Animal Care and Use Committee.

Note that full information on the approval of the study protocol must also be provided in the manuscript.

Human research participants

Policy information about studies involving human research participants

Population characteristics	Research participants who provided Blood and aqueous humor samples were from Indian decent. All Post-mortom eyes from AMD patients and age-matched controls were from Caucasian decent. The detailed characteristics for the population is provided in the methods section and Table S1.
Recruitment	Participants were chosen by Ophthalmic examination to identify AMD patients as explained in the methods section. Post-mortom eyes were grossly evaluated by microscopy to identify AMD patients and patient history was obtained from eye bank. Subjects with co-existing glaucoma or any other degenerative retinal disorders were excluded. The control group consisted of individuals without any history of AMD, diabetes, cardiovascular disorders or retinal diseases.
Ethics oversight	All patient samples (blood and aqueous humor) and related clinical information were collected after obtaining approval by the Narayana Nethralaya Institutional Review Board (IRB) and with written, informed consent from patients.

Note that full information on the approval of the study protocol must also be provided in the manuscript.

Flow Cytometry

Plots

Confirm that:

- [x] The axis labels state the marker and fluorochrome used (e.g. CD4-FITC).
- [x] The axis scales are clearly visible. Include numbers along axes only for bottom left plot of group (a 'group' is an analysis of identical markers).
- [x] All plots are contour plots with outliers or pseudocolor plots.
- [x] A numerical value for number of cells or percentage (with statistics) is provided.

Methodology

Sample preparation	The detailed sample preparation for all flow cytometry experiments are described in the methods section.
Instrument	BD FACS ARIA III, BD FACS Canto II.
Software	BD FACS DIVA, FlowJo (Version 7.6.5).
Cell population abundance	Sorting was not performed in this study.
Gating strategy	The gating strategy is described in the methods section and in the Figure 1 (mouse retina samples) and supplementary figure 2 (human blood samples)

- [x] Tick this box to confirm that a figure exemplifying the gating strategy is provided in the Supplementary Information.
